# Effects of coronavirus disease 2019 (COVID-19) pandemic on antimicrobial prevalence and prescribing in a tertiary hospital in Singapore

**DOI:** 10.1186/s13756-021-00898-8

**Published:** 2021-02-03

**Authors:** Tat Ming Ng, Sock Hoon Tan, Shi Thong Heng, Hui Lin Tay, Min Yi Yap, Boon Hou Chua, Christine B. Teng, David C. Lye, Tau Hong Lee

**Affiliations:** 1 Department of Pharmacy, Tan Tock Seng Hospital, Singapore, Singapore; 2 Department of Pharmacy, Faculty of Science, National University of Singapore, Singapore, Singapore; 3 Department of Infectious Diseases, National Centre for Infectious Diseases, Singapore, Singapore; 4 Department of Infectious Diseases, Tan Tock Seng Hospital, Singapore, Singapore; 5 Lee Kong Chian School of Medicine, Nanyang Technological University, Singapore, Singapore; 6 Yong Loo Lin School of Medicine, National University of Singapore, Singapore, Singapore

**Keywords:** COVID-19, Antimicrobial prevalence, Singapore, Resources, Antimicrobial stewardship, Pandemic

## Abstract

**Background:**

The deployment of antimicrobial stewardship (AMS) teams to deal with the COVID-19 pandemic can lead to a loss of developed frameworks, best practices and leadership resulting in adverse impact on antimicrobial prescribing and resistance. We aim to investigate effects of reduction in AMS resources during the COVID-19 pandemic on antimicrobial prescribing.

**Methods:**

One of 5 full-time equivalent AMS pharmacists was deployed to support pandemic work and AMS rounds with infectious disease physicians were reduced from 5 to 2 times a week. A survey in acute inpatients was conducted using the Global Point Prevalence Survey methodology in July 2020 and compared with those in 2015 and 2017–2019.

**Results:**

The prevalence of antimicrobial prescribing (55% in 2015 to 49% in 2019 and 47% in 2020, p = 0.02) and antibacterials (54% in 2015 to 45% in 2019 and 42% in 2020, p < 0.01) have been reducing despite the pandemic. Antimicrobial prescribing in infectious disease wards with suspected or confirmed COVID-19 cases was 29% in 2020. Overall, antimicrobial prescribing quality indicators continued to improve (e.g. reasons in notes, 91% in 2015 to 94% in 2019 and 97% in 2020, p < 0.01) or remained stable (compliance to guideline, 71% in 2015 to 62% in 2019 and 73% in 2020, p = 0.08).

**Conclusion:**

During the COVID-19 pandemic, there was no increase in antimicrobial prescribing and no significant differences in antimicrobial prescribing quality indicators.

## Background

The global response to the coronavirus disease 2019 (COVID-19) pandemic has focused on controlling the spread of infection and development of treatment and vaccines [1]. The typical symptoms of patients with severe acute respiratory syndrome coronavirus 2 (SARS-CoV-2) infection are fever, sore throat, fatigue, cough or dyspnea [2]. These symptoms may prompt clinicians to start antibiotics to treat community-acquired pneumonia or COVID-19 with secondary bacterial infection. In a review of patients with coronavirus infections, 62/806(8%) of patients with COVID-19 were reported to have bacterial or fungal co-infections. However, among COVID-19 patients, 1450 of 2010 (72%) received antibiotics. These agents tended to be broad-spectrum and empiric [3]. As the pandemic continues, treatment of patients with respiratory symptoms can drive increasing rates of empiric antimicrobial therapy.

Antimicrobial stewardship (AMS) teams generally comprise infectious disease physicians, AMS pharmacists or pharmacists with special interest in infectious diseases. Many of these individuals are re-deployed to focus on patient care and other areas directly related to COVID-19 during the pandemic. This can lead to loss of established AMS frameworks, best practices, and leadership in healthcare institutions, resulting in adverse impact on antimicrobial resistance in the long term [4]. It is important that healthcare systems keep AMS in consideration as they channel resources to control COVID-19 [5].

The Global Point Prevalence Survey (Global-PPS) first conducted in 2015 provides a rapid way to understand the quantity and quality of antimicrobial prescribing [6]. Using a standardised methodology over the years, the surveys allow comparisons to be made between time periods [7]. Information from these surveys can be used for tailor made surveillance and help develop prescribing guidelines and educational initiatives to improve antimicrobial use. The antimicrobial stewardship team at Tan Tock Seng Hospital (TTSH) and the National Centre for Infectious Diseases (NCID), Singapore, comprise a team of 5 full-time equivalent (FTE) pharmacists who provided daily prospective review and feedback (PRF) on piperacillin-tazobactam, carbapenem and ciprofloxacin use. They work closely with a team of 5 Infectious diseases (ID) physicians who take turns to perform PRF on complex cases. Since the start of the pandemic in January 2020, 1 full-time equivalent of AMS pharmacist was deployed to support COVID-19 clinical trials and only 1–2 ID physicians continued with PRF once to twice a week. The other ID physicians were deployed to duties related to the pandemic.

In this study, we aimed to compare the prevalence of antimicrobial use and quality indicators in hospitalised patients using the Global-PPS methodology from 2015 and 2017–2020 to investigate the effects of the deployment of AMS manpower resources to support the ongoing COVID-19 efforts.

## Methods

### Study design and setting

This was a retrospective study comparing 5 cross-sectional surveys conducted in years 2015, 2017, 2018, 2019 and 2020. The survey methodology was in accordance with Global-PPS study protocol in 2015 and 2017 -2020. They were conducted in acute care wards of TTSH, a 1500-bed university teaching adult hospital in Singapore, in the 5 years. The new 330-bed NCID, located in the same campus, was included in years 2019 and 2020. NCID, is a purpose-built infectious disease management facility. During the COVID-19 pandemic, high risk suspect cases were admitted, isolated and tested for SARS-CoV-2. Low risk suspect cases were tested and discharged to self-isolate at home. Phone surveillance for symptom progression was performed and patients with persistent symptoms or positive swab results were recalled for further evaluation or isolation. Close contacts of confirmed COVID-19 patients were identified and place in quarantine either at home or government quarantine facilities while casual contacts were placed o phone surveillance [8]. The Global-PPS was conducted on any day except weekends and public holidays by the AMS pharmacists. The 5 surveys were conducted in March 2015, November 2017, October 2018, April 2019 and July 2020. All inpatients were included for audit at 0800 h of the chosen survey day and the survey was completed over several days. Following the Global-PPS annual protocols, antimicrobials for topical use were excluded.

### Data

Data collection was completed using Global-PPS methodology specified forms. The ward form included the total number of admitted inpatients at 0800 h on survey day and the total number of available inpatient beds in the surveyed wards. The patient form was only completed if patient included in the survey was on at least 1 systemic antimicrobial at 0800 h on survey day. Patient data was collected using electronic medical records. No patient identifier was collected. The survey was completed without questioning the diagnoses indicated by medical teams. The type of data collected were described previously (https://www.global-pps.com/) [6]. Specifically, age, ward type, type of antimicrobial, diagnosis, type of indication (i.e. whether prophylactic use, or for community-onset or healthcare-associated infections), whether treatment was empiric or targeted, and quality indicators of antimicrobial prescribing were collected. Compliance to institutional guidelines was only reported for empiric use and surgical prophylaxis. Quality indicators of antimicrobial use considered documented reason for antimicrobial treatment, and presence of stop/review dates. Hospital antimicrobial utilization was also collected for the period before and during COVID-19 pandemic for changes in antimicrobial prescribing behaviours. Utilization data was extracted from pharmacy dispensing records between January 2019 and September 2020, including April 2020, which was the peak of the COVID-19 pandemic in Singapore [9]. All antimicrobials excluding antituberculosis drugs, antiretrovirals and antimalarials were monitored. Daily defined dose (DDD) was used as the measurement unit for antimicrobial utilization, which was adjusted for every 1000 patient days per month.

The Global-PPS protocol remained consistent over the years with minor changes. In 2018, antivirals other than neuraminidase inhibitors were added. The other changes were described in the Additional file 1. The study was approved by local institutional review board (DSRB reference: 2015/00015, 2017/01012, 2019/00768, 2020/01045).

### Statistical analysis

Comparisons of proportions for binary variables were performed using chi-squared test for trend in proportions where appropriate. The median of continuous variables was compared using the Kruskal–Wallis test. A *P*-value of < 0.05 was used as the level of significance. The statistical tests were performed using R software version 3.5.0. Segmented regression analysis was performed to describe the utilization trends before and after the peak of the pandemic. The Stata package ITSA was used and the statistics were estimated using ordinary least squares regression and Newey-West regression was specified to account for an error structure that is assumed to be heteroskedastic and autocorrelated at lag 0. The autocorrelation of each model was tested using Cumby–Huizinga general test and visual inspection of autocorrelation and partial autocorrelation plots.

## Results

**Fig. 1:**
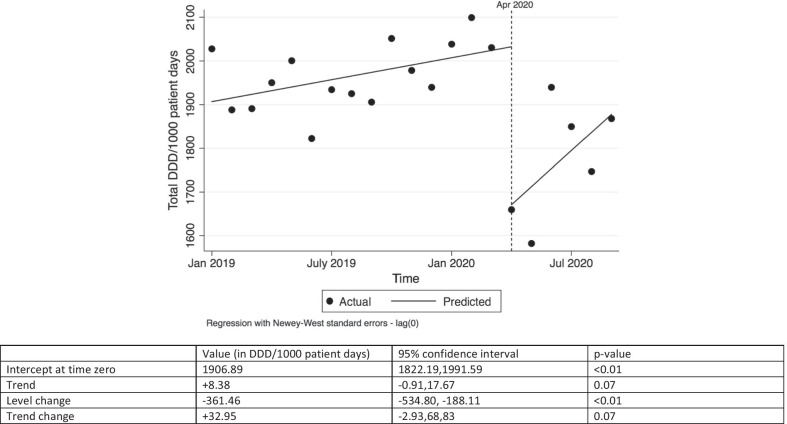
Segmented regression analysis of monthly total antimicrobial utilization in the hospital from January 2019 till September 2020, in daily defined dose (DDD) per 1000 patient days (April 2020 was observed as the peak of COVID-19 pandemic in Singapore)

The first COVID-19 case was cared for in our facility on 2^nd^ February 2020. On 15 July 2020, there were 65 inpatients with confirmed COVID-19 and 64 new admissions for suspected COVID-19. A cumulative total of 8952 COVID-19 patients had been admitted to our facility, of which 16 had died. Another 6105 admissions had been investigated for suspected COVID-19. In terms of antimicrobial utilization, there was a significant reduction of 361.46 DDD/1000 patient days/month (p < 0.01) before and after the peak of the COVID-19 pandemic in Singapore in April 2020 [9]. At the point of PPS survey (July 2020), the total antimicrobial utilization was 1849.71 DDD/1000 patient days/month. (Fig. 1).

The Global-PPS dataset from 2015 and 2017- 2020 included all acute inpatient wards. The median number of available beds on survey day annually was 1357 (range 1135–1375). The median number of patients admitted was 1173 (range 1150–1214). The median number of adult wards surveyed annually was 44 (range 35–50). On the survey day in July 2020, 4 surgical and 2 medical wards were closed due to COVID-19; 2 intensive care wards (ICU) and 9 infectious disease wards (AMW-ID) catered to 199 suspected and confirmed COVID-19 patients. Among these patients, 15 were admitted to ICU and 184 were managed as medical patients in the infectious disease wards. Overall number of admitted patients were similar but the proportion of surgical patients were lower in 2020 (19%) compared to previous years (range 22–27%).

There was a median of 780 (range 755–839) antimicrobial prescriptions on the survey day. Antibacterials for systemic use, corresponding to World Health Organization anatomical therapeutic chemical (WHO ATC) code J01, accounted for 84% of total prescriptions with a median of 665 (range 615–727) on survey day. Antimycotic (ATC code J02) prescriptions accounted for 2% of total prescriptions with a median of 18 (range 11–20) prescriptions, antimycobacterials (ATC code J04) accounted for 6% of total prescriptions with a median of 50 (range 21–52) on survey day. Intestinal anti-infectives (ATC code A07A) and antivirals for systemic use (ATC code J05) accounted for 1% and 7% of total prescriptions and median of 6 (range 1–14) and 70 (range 8–83) prescriptions on survey day. Overall, the mean prevalence of patients on antimicrobials was 49% (range, 45–55%) and varied significantly over the years. It has reduced from 55% in 2015 to 49% in 2019 and 47% in 2020 (p-value of test for trend in proportions = 0.02). Similar trends were observed in terms of antibacterials for systemic use (ATC code J01) from 54% in 2015 to 45% in 2019 and 42% in 2020 (p-value of test for trend in proportions < 0.01) (Table 1).

**Table 1:** Antimicrobial use in inpatients from 2015 to 2019

Year	2015	2017	2018	2019	2020	P
No. of available beds (No. of surveyed wards)	1135 (35)	1345 (40)	1359 (44)	1375 (46)	1357 (50)	–
No. of patients admitted	1018	1150	1173	1214	1182	NA
No. of patients on antimicrobials	558 (55%)	519 (45%)	592 (50%)	589 (49%)	558 (47%)	0.02
No. of patients on antibacterials for systemic use (J01)	550 (54%)	504 (44%)	562 (48%)	550 (45%)	501 (42%)	< 0.01
Age, median years (range)	73 (16–99)	69 (17–100)	71 (17–100)	72 (15–99)	71 (16–101)	–
Male	290 (53%)	281 (54%)	340 (57%)	345 (59%)	330 (59%)	–
Medical patients	704 (69%)	802 (70%)	849 (72%)	914 (75%)	912 (77%)	< 0.01
Surgical patients	278 (27%)	297 (26%)	290 (25%)	273 (22%)	226 (19%)	< 0.01
Intensive care patients	36 (4%)	51 (4%)	34 (3%)	27 (2%)	44 (4%)	0.27
No. of antimicrobials	768	755	839	821	780	–
Antibacterials for systemic use (J01)	727 (95%)	647 (86%)	692 (82%)	665 (81%)	615 (79%)	–
Antimalarials (P01BA)	0	2 (0.2%)	0	10 (1%)	4 (0.5%)	–
Antimycobacterials (J04)	21 (3%)	51 (7%)	50 (6%)	44 (5%)	52 (7%)	–
Antimycotics for systemic use (J02)	11 (1%)	19 (3%)	13 (2%)	18 (2%)	20 (3%)	–
Antivirals for systemic use (J05)	8 (1%)	26 (3%)	80 (10%)*	70 (9%)	83 (11%)	–
Intestinal anti-infectives (A07A)	1 (< 1%)	10 (1%)	4 (< 1%)	14 (2%)	6 (< 1%)	–

P = test for trends in proportions and test for differences in median using the Kruskal–Wallis test as appropriate

^*^Introduction of the surveillance of the complete list of antivirals for systemic use (J05) as opposed to surveillance of neuraminidase inhibitors (J05AH) alone in 2015 and 2017

Overall, the prevalence of patients on antimicrobials was reducing but not significantly different in the medical (56% in 2015 to 47% in 2020) and haematology-oncology wards (73%, 2015 to 66%, 2020) (p-value of test for trend in proportions > 0.05). The proportion of patients on antimicrobials increased significantly in the surgical wards (49%, 2015 to 58%, 2020) (p-value of test for trend in proportions = 0.03). In intensive care wards, proportion of patients on antimicrobials decreased from 2015 to 2018 but increased to a high of 81% in 2019 (p-value of test for trend in proportions = 0.18). In 2020, antimicrobial use in the infectious disease wards which housed suspected or confirmed COVID-19 cases was 29%. (Table 2).

**Table 2:** Patients on antimicrobials divided by ward types from 2015 to 2020

Year	2015	2017	2018	2019	2020	P
Overall	558/1018 (55%)	519/1150 (45%)	592/1173 (50%)	589/1214 (49%)	558/1182 (47%)	0.02
Medical ward	331/596 (56%)	335/780 (43%)	404/852 (47%)	395/860 (46%)	372/789 (47%)	0.08
Surgical ward	168/341 (49%)	133/281 (47%)	139/244 (57%)	151/285 (53%)	76/130 (58%)	0.03
Intensive care ward	32/44 (73%)	30/51 (59%)	23/41 (56%)	26/32 (81%)	34/44 (75%)	0.18
Haematology and oncology ward	27/37 (73%)	21/38 (55%)	26/36 (72%)	17/37 (46%)	23/35 (66%)	0.34
Infectious disease ward	–	–	–	–	53/184 (29%)	–

P = test for trends in proportions

Over the years, common indications for antimicrobial use were community acquired infections (47–63%) and healthcare-associated infections (26–39%). The top reasons for starting antibiotics were pneumonia or lower respiratory tract infections (26–34%), skin and soft tissue infections (11–15%), intra-abdominal infections (9–10%), lower urinary tract infections (4–12%), and upper urinary tract infections (3–13%) (Table 3).

**Table 3:** Indications of antimicrobials use and top 10 reasons to treat inpatients with at least one antibiotic for systemic use (J01), year 2015–2020

Year	2015	2017	2018	2019	2020
	No. of antimicrobials				
Type of indication	768	755	839	821	780
Community acquired infections	482 (63%)	353 (47%)	500 (60%)	487 (59%)	444 (57%)
Healthcare associated infection	227 (30%)	295 (39%)	245 (29%)	212 (26%)	247 (32%)
Medical prophylaxis	8 (1%)	30 (4%)	37 (4%)	35 (4%)	42 (5%)
Surgical prophylaxis	36 (5%)	34 (5%)	26 (3%)	26 (3%)	27 (4%)
Unknown indication	13 (2%)	39 (5%)	23 (3%)	28 (3%)	13 (2%)
Others (e.g. use as prokinetic)	2 (< 1%)	4 (1%)	8 (1%)	33 (4%)	7 (1%)
	No. of patients				
Top 10 diagnosis	550	504	562	550	501
Pneumonia or lower respiratory tract infection	184 (34%)	142 (29%)	144 (26%)	150 (27%)	144 (29%)
Skin and soft tissue infections†	81 (15%)	54 (11%)	61 (11%)	75 (14%)	66 (13%)
Intra-abdominal infections^‡^	54 (10%)	47 (9%)	55 (10%)	49 (9%)	44 (9%)
Upper urinary tract infections^§^	28 (5%)	14 (3%)	61 (11%)	48 (9%)	65 (13%)
Lower urinary tract infections (cystitis)	44 (8%)	58 (12%)	44 (8%)	36 (7%)	20 (4%)
Gastrointestinal infections	16 (3%)	23 (5%)	21 (4%)	24 (4%)	21 (4%)
Unknown	8 (2%)	36 (7%)	26 (5%)	21 (4%)	10 (2%)
Bone and joint infections**	12 (2%)	14 (3%)	19 (3%)	22 (4%)	17 (3%)
Acute Bronchitis or exacerbations of chronic bronchitis	10 (2%)	15 (3%)	17 (3%)	17 (3%)	6 (1%)
Sepsis^††^	19 (4%)	23 (5%)	12 (2%)	15 (3%)	8 (2%)

Patients recorded with more than one diagnosis were counted by number of diagnoses

Patients not treated with antibiotics for systemic use, but who were treated with other antimicrobials (e.g., antimalarials) were not included

^†^Includes cellulitis, wound infections (including surgical site infections), deep soft tissue infections not involving bone (e.g., infected pressure or diabetic ulcers, abscesses). ‡Includes intra-abdominal sepsis and hepatobiliary and intra-abdominal abscesses. §Includes catheter-related urinary tract infections and pyelonephritis

**Includes septic arthritis (including prosthetic joints) and osteomyelitis

^††^Includes sepsis syndrome or septic shock with no clear anatomical site

**Fig. 2:**
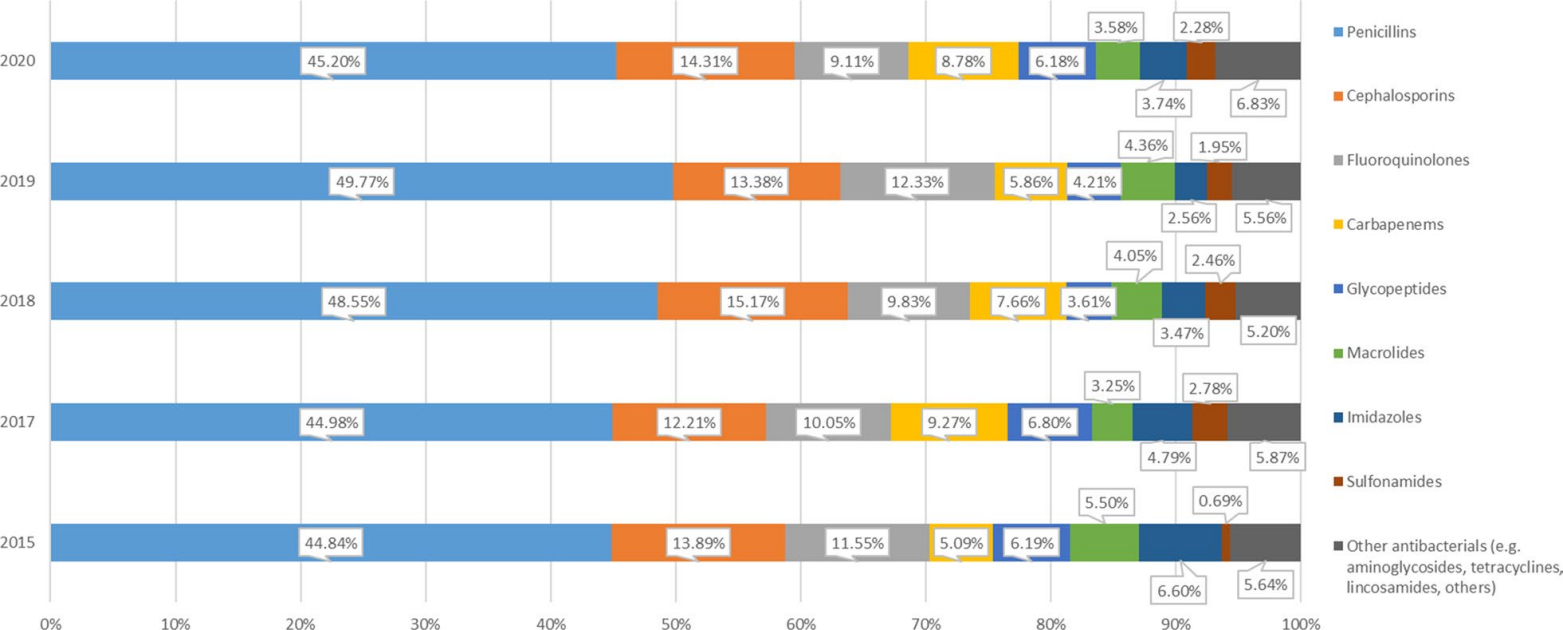
Anti-bacterial use from 2015 to 2020

Forty-seven different systemic antibacterials were used in patients admitted to adult wards on the survey days. The penicillins were the most prescribed class (1562/3346 antibacterial prescriptions, 47%), comprising mainly amoxicillin with beta-lactamase inhibitor (1125/3346, 34%) and piperacillin with beta-lactamase inhibitor (300/3346, 9%). The second and third most prescribed antibacterials were cephalosporins (462/3346,14%)—mainly cefazolin, ceftriaxone, and fluoroquinolones (355/3346, 11%)- mainly ciprofloxacin and levofloxacin. The overall antibacterial utilisation trends were stable since 2015 (Fig. 2). Amoxicillin with beta-lactamase inhibitor proportion increased from 28.2% in 2015 to 37.0% in 2017, then decreased to 36.1% in 2019 and 34.8% in 2020. Piperacillin with beta-lactamase inhibitor proportion was on downward trend from 10.7% in 2015 to 9.2% in 2019 and 7.0% in 2020. Carbapenem use increased from 5.1% in 2015 to 9.3% in 2017, then decreased to 5.9% in 2019 and 8.8% in 2020, this change was mainly driven by Meropenem (3.9% in 2015 to 5.1% in 2019 and 7.6% in 2020). Ciprofloxacin proportion changed from 8.3% in 2015 to 9.3% in 2019 and 5.0% in 2020.

For quality indicators, there was an overall improvement in terms of documented reasons for giving antimicrobials in the medical records: 91% in 2015 to 94% in 2019 and 97% in 2020 (p-value of test for trend in proportions < 0.01); this trend was similarly observed in surgical patients (Additional file 1: Table 2). The documentation of stop/review date improved from 53% in 2015 to 56% in 2019 and 61% in 2020 (p-value of test for trend in proportions < 0.01). Overall compliance to guideline did not change significantly from 71% in 2015 to 62% in 2019 and 73% in 2020 (p-value of test for trend in proportions = 0.08); the compliance rate did not significantly change over the years among surgical patients (p-value of test for trend in proportions = 0.493), medical patients (p-value of test for trend in proportions = 0.435), and intensive care patients (p-value of test for trend in proportions = 0.056) respectively (Additional file 1: Tables 1–3). The proportion of surgical antimicrobial prophylaxis prescriptions being ordered for more than 1 day did not significantly decrease over time: 56% in 2015 to 69% in 2019 and 52% in 2020 (p-value of test for trend in proportions = 0.76) (Table 4).

**Table 4:** Empiric antimicrobial use and prescribing quality indicators from 2015 to 2020

Year	2015	2017	2018	2019	2020	P
No. of anti-microbials	768	755	839	821	780	–
Empiric treatment	608 (79%)	538 (71%)	660 (79%)	577 (70%)	532 (68%)	–
Reasons in notes	697 (91%)	733 (97%)	746 (89%)	772 (94%)	755 (97%)	< 0.01
Stop/review date	404 (53%)	323 (43%)	450 (54%)	458 (56%)	476 (61%)	< 0.01
Guideline compliant^a^	340/479 (71%)	291/375 (78%)	286/403 (71%)	235/377 (62%)	248/344 (73%)	0.08
No guideline available	96/589 (16%)	66/480 (14%)	138/560 (25%)	108/507 (21%)	110/463 (24%)	–
Surgical prophylaxis > 24 h	20/36 (56%)	20/34 (59%)	11/26 (42%)	18/26 (69%)	15/27 (52%)	0.76

^a^The number of antimicrobial prescriptions for which guidelines were available was used as the denominator to calculate percentages. Only includes empiric and surgical prophylaxis use. P = test for trends in proportions

The number of courses of carbapenems, piperacillin-tazobactam and ciprofloxacin reduced by 17% from 7046 during the 6-month period from July to December 2019 to 5852 during the next 6-month period from January to June 2020. However, the proportion of courses reviewed by AMS team were maintained at 4834/7046 (69%) compared to 4141/5853 (71%). The number of recommendations made and accepted were also maintained during both periods, 1151/1440 (80%) vs. 1204/1537 (78%).

## Discussion

Despite the re-deployment of our AMS manpower, the prevalence of antimicrobial and antibacterial use did not increase despite the COVID-19 pandemic. Antimicrobial utilization was very much lower at the peak of the COVID-19 pandemic as hospital resources pivoted to taking care of COVID-19 patients. In 2020, about a quarter of patients in the infectious disease wards were on antimicrobials. There were fewer surgical patients but there was a higher proportion of antibiotic use among them in 2020 due to the cancellation of elective and postponement of non-urgent procedures. Therefore, surgical inpatients could have been sicker and more often required antibiotics.

Among the antibacterials audited via prospective review and feedback, only meropenem prevalence showed an increase. However, this trend started before the pandemic. Overall, antimicrobial prescribing quality indicators continued to improve or remained stable. We found that only 29% of patients with suspected or confirmed COVID-19 were on antibacterial agents. This is in stark contrast to 71.9%, reported by Langford et al. in a recently published meta-analysis and 71% reported by Nori et al. during the March to May pandemic surge period in New York [10, 11]. This may be accounted by rapid confirmatory diagnosis of SARS-CoV-2 infection as the cause of pneumonia in our setting, and the vast majority of patients having non-severe infections [12]. To prepare for the pandemic, the AMS team was requested by the hospital management to work with hospital pharmacy to provide recommendations on alternative antimicrobial use in anticipation of possible drug shortfall. In addition, the team briefed the hospital senior management on the possibilities of antibiotic supply disruption and shared best practices and common inappropriate antibiotic use previously observed during audit and feedback. These case vignettes were shared with all doctors and pharmacists via monthly email messages. It is possible that these activities reminded the prescribers on the ground to be more judicious in antimicrobial use.

The sustainable culture of judicious antimicrobial use was developed over the years at our institutions. This could have contributed to continued practice of appropriate prescribing. Since 2009, our multi-disciplinary AMS programme introduced guidelines and performed prospective review and feedback. These measures were augmented by computerised clinical decision support systems (introduced in 2011) and educational efforts to engage healthcare providers and the public on appropriate use of antimicrobials. [13–15] Results of the PPS were shared with the hospital management and senior doctors to raise awareness on the high prevalence of antimicrobial prescribing. These activities may have driven the improvement in some antimicrobial prescribing quality indicators and reduced the prevalence of antimicrobials among acute inpatients. Collectively, the AMS efforts over the years could have paid dividends during the time of COVID-19 pandemic. The maturity of antimicrobial prescribing habits may have sustained the practices even when AMS resources were substantially reduced.

While the AMS team reduced in size, the team continued prospective review and feedback during the pandemic, focussing on meropenem, piperacillin-tazobactam and oral ciprofloxacin use. The smaller team of pharmacists and physicians maintained the review rate of these antibiotics and provided comparable number of recommendations with similar acceptance rate. Although these constituted a small proportion of the total antimicrobials used, the continued presence of the AMS team and the recommended interventions may have encouraged prescribers to maintain judicious use of antibiotics.

As these were point prevalence studies, variation within and between survey periods may not be sufficiently accounted for. The appropriateness of antimicrobial duration and the impact of duration of therapy on antimicrobial utilisation were not reported. The compliance to guidelines was assessed solely based on the prescribing doctor’s documented diagnosis or reasons of use. Other opportunities for improved antimicrobial prescribing such as dose, route and duration were not assessed.

## Conclusion

As COVID-19 diverted resources from AMS teams, there was no significant deterioration in trends of antimicrobial use or reduction in quality of antimicrobial prescribing at our institutions. Despite a smaller AMS team, the presence of an established multi-disciplinary AMS programme prior to the COVID-19 pandemic managed to keep antimicrobial prevalence and quality of antimicrobial prescribing stable in our institutions. As the pandemic continues, attention must be given to control the amount and appropriateness of antimicrobial use. AMS resources and efforts should be enhanced especially in areas where AMS practices are still in early stages of development. As the global momentum of controlling antimicrobial resistance accumulate in last few years, careful and deliberate actions must be taken now so that the COVID-19 pandemic does not derail this process.

## Supplementary Information

**Additional file 1.** Percentage of Empiric antimicrobial use and prescribing quality indicators for medical, surgical and intensive care patients respectively from 2015 to 2020.

## Data Availability

The datasets used and/or analysed during the current study are available from the corresponding author on reasonable request.

## Abbreviations

COVID-19: Coronavirus disease 2019
AMS: Antimicrobial stewardship
TTSH: Tan Tock Seng Hospital
NCID: National Centre for Infectious Diseases
FTE: Full-time equivalent
ID: Infectious diseases
Global-PPS: Global Point Prevalence Survey
