# Supplementary material for: Effects of coronavirus disease 2019 (COVID-19) pandemic on antimicrobial prevalence and prescribing in a tertiary hospital in Singapore
Source: Antimicrob Resist Infect Control. 2021 Feb 3;10:28. doi: 10.1186/s13756-021-00898-8 (PMC7856610; doi:10.1186/s13756-021-00898-8)
Supplement: Supplementary file 1 — Additional file 1. Percentage of Empiric antimicrobial use and prescribing quality indicators for medical, surgical and intensive care patients respectively from 2015 to 2020. [file 13756_2021_898_MOESM1_ESM.docx]

Appendix

Table 1. Empiric antimicrobial use and prescribing quality indicators for medical patients from 2015 to 2020

| Year | 2015 | 2017 | 2018 | 2019 | 2020 | P |
| --- | --- | --- | --- | --- | --- | --- |
| No. of anti-microbials | 538 | 515 | 601 | 602 | 559 | - |
| Empiric treatment | 442 (82%) | 388 (75%) | 487 (81%) | 419 (70%) | 364 (65%) | - |
| Reasons in notes | 519 (96%) | 499 (97%) | 555 (92%) | 576 (96%) | 546 (98%) | 0.617 |
| Stop/review date | 299 (56%) | 230 (45%) | 334 (56%) | 348 (58%) | 357 (64%) | <0.01 |
| Guideline compliant^a^ | 266/338 (79%) | 201/256 (79%) | 205/284 (72%) | 205/284 (72%) | 170/228 (75%) | 0.056 |
| No guideline available | 80/426 (19%) | 51/334 (15%) | 109/395 (28%) | 74/353 (21%) | 74/306 (24%) | - |
| Surgical prophylaxis > 24 hours | 0/0  (0%) | 0/1  (0%) | 0/0  (0%) | 1/1  (100%) | 1/2  (50%) | - |

^a^The number of antimicrobial prescriptions (ATC code J01) for which guidelines were available was used as the denominator to calculate percentages. Only includes empiric and surgical prophylaxis use. ^b^The total number of antimicrobial prescriptions (ATC code J01) for empiric/surgical prophylaxis therapy was used as the denominator to calculate percentages. P= test for trends in proportions.

Table 2. Empiric antimicrobial use and prescribing quality indicators for surgical patients from 2015 to 2020

| Year | 2015 | 2017 | 2018 | 2019 | 2020 | P |
| --- | --- | --- | --- | --- | --- | --- |
| No. of anti-microbials | 194 | 190 | 200 | 179 | 161 | - |
| Empiric treatment | 145 (75%) | 123 (65%) | 147 (74%) | 127 (71%) | 114 (71%) | - |
| Reasons in notes | 143 (74%) | 186 (98%) | 156 (78%) | 157 (88%) | 152 (94%) | <0.01 |
| Stop/review date | 80 (41%) | 79 (42%) | 93 (47%) | 91 (51%) | 87 (54%) | <0.01 |
| Guideline compliant^a^ | 59/123 (48%) | 73/98 (74%) | 62/97 (64%) | 38/79 (48%) | 47/74 (64%) | 0.493 |
| No guideline available^b^ | 16/143 (11%) | 13/120 (11%) | 26/140 (19%) | 32/124 (26%) | 32/110 (29%) | - |
| Surgical prophylaxis > 24 hours | 20/35  (57%) | 20/32  (63%) | 11/22  (50%) | 16/22  (73%) | 12/22  (55%) | <0.01 |

^a^The number of antimicrobial prescriptions (ATC code J01) for which guidelines were available was used as the denominator to calculate percentages. Only includes empiric and surgical prophylaxis use. ^b^The total number of antimicrobial prescriptions (ATC code J01) for empiric/surgical prophylaxis therapy was used as the denominator to calculate percentages. P= test for trends in proportions.

Table 3. Empiric antimicrobial use and prescribing quality indicators for intensive care patients from 2015 to 2020

| Year | 2015 | 2017 | 2018 | 2019 | 2020 | P |
| --- | --- | --- | --- | --- | --- | --- |
| No. of anti-microbials | 36 | 50 | 38 | 40 | 60 | - |
| Empiric treatment | 21 (58%) | 27 (54%) | 26 (68%) | 31 (78%) | 54 (90%) | - |
| Reasons in notes | 35 (97%) | 48 (96%) | 35 (92%) | 39 (98%) | 57 (95%) | 0.770 |
| Stop/review date | 25 (69%) | 14 (28%) | 23 (61%) | 19 (48%) | 32 (53%) | 0.988 |
| Guideline compliant^a^ | 15/18 (83%) | 17/21 (81%) | 19/22 (86%) | 25/28 (89%) | 31/42 (74%) | 0.435 |
| No guideline available^b^ | 0/20 (0%) | 2/26 (8%) | 3/25 (12%) | 2/30 (7%) | 4/47 (9%) | - |
| Surgical prophylaxis > 24 hours | 0/1  (0%) | 0/1  (0%) | 0/4  (0%) | 1/2  (50%) | 1/3  (33%) | - |

^a^The number of antimicrobial prescriptions (ATC code J01) for which guidelines were available was used as the denominator to calculate percentages. Only includes empiric and surgical prophylaxis use. ^b^The total number of antimicrobial prescriptions (ATC code J01) for empiric/surgical prophylaxis therapy was used as the denominator to calculate percentages. P= test for trends in proportions.
